# Characteristics of ^18^F-FAPI-04 PET/CT in patients with peritoneal metastasis and to predict treatment efficacy, a head-to-head comparison with ^18^F-FDG PET/CT

**DOI:** 10.1186/s40644-025-00887-9

**Published:** 2025-06-02

**Authors:** Yafei Zhang, Mimi Xu, Yu Wang, Fang Yu, Xinxin Chen, Guangfa Wang, Kui Zhao, Hong Yang, Xinhui Su

**Affiliations:** 1 Department of Nuclear Medicine, The First Affiliated Hospital, Zhejiang University School of Medicine, 79 Qingchun Road, Hangzhou, 310003 China; 2 Department of Pharmacy, The Second Affiliated Hospital of Zhejiang, Chinese Medical University, Hangzhou, 310005 China; 3 Department of Pathology, The First Affiliated Hospital, Zhejiang University School of Medicine, Hangzhou, 310003 China; 4 Department of Radiology, The First Affiliated Hospital, Zhejiang University School of Medicine, 79 Qingchun Road, Hangzhou, 310003 China

**Keywords:** Peritoneal metastases, Fibroblast activation protein, ^18^F-FAPI-04, PET/CT

## Abstract

**Background:**

^18^F-FAPI-04 PET/CT shows promise in detecting peritoneal metastases (PM), but its superiority over ^18^F-FDG PET/CT for lesion detection and predicting chemotherapy benefit remains unclear.

**Purpose:**

To compare ^18^F-FAPI-04 and ^18^F-FDG PET/CT imaging features in PM and assess predictive value of ^18^F-FAPI-04 for chemotherapy efficacy.

**Methods:**

39 pathologically confirmed PM patients with digestive malignancies underwent concurrent ^18^F-FAPI-04 and ^18^F-FDG PET/CT. Semi-quantitative parameters, including SUV_max_, tumor/liver ratio (T/L), tumor/mediastinal blood pool ratio (T/B), were analyzed. The tracer uptake was compared via Wilcoxon tests. The relationships between ^18^F-FAPI-04 uptake with FAP and α-SMA expression were analyzed using Pearson correlation. Patients were divided into different short-term outcome groups (responders vs. non-responders) according to RECIST criteria (v.1.1) after chemotherapy. Post-chemotherapy outcomes were evaluated using logistic regression.

**Results:**

Patients (median age 62; 16 females, 23 males) included pancreatic (*n* = 17), cholangiocarcinoma (*n* = 8), gastric (*n* = 6), and colorectal cancers (*n* = 8). ^18^F-FAPI-04 demonstrated significantly higher SUV_max_, T/L, and T/B than ^18^F-FDG (*P* < 0.05). Pancreaticobiliary cancers (pancreatic/cholangiocarcinoma) exhibited higher 18F-FAPI-04 uptake than gastroenteric cancers (gastric/colorectal) (*P* < 0.05), though no differences existed within subgroups. ^18^F-FAPI-04 parameters positively correlated with FAP and α-SMA expression. In univariate analysis, ^18^F-FAPI-04 uptake differed significantly between responders and non-responders. Multivariate analysis identified SUV_max_ as an independent predictor (OR = 1.354, 95%CI:1.025–1.788, *P* = 0.033). Optimal ^18^F-FAPI-04 cut-offs for distinguishing outcomes were SUV_max_=11.05 (AUC = 0.783; sensitivity = 70.60%, specificity = 80.40%), T/L = 7.53 (AUC = 0.717; 58.82%, 81.82%), and T/B = 8.76 (AUC = 0.751; 64.71%, 86.37%).

**Conclusion:**

^18^F-FAPI-04 PET/CT outperforms ^18^F-FDG in PM detection, with semi-quantitative parameters predicting chemotherapy response.

## Background

Peritoneal metastasis (PM) is not uncommon in advanced stages of abdominal malignancies, such as colorectal, gastric, cholangiocarcinoma, and pancreatic cancer [1–4] and is a common cause of mortality, with median survival period of 6–12 months without treatment [5]. Over the years, an increase in patients with PM has been reported in all described tumor types, which can be primarily attributed to better diagnostic techniques [6, 7]. However, the currently reported incidence may remain underestimated due to the challenging diagnosis of PM by imaging techniques and the possible inaccurate reporting of metastatic sites in cases of extensive disease [1]. Although diagnostic laparoscopy is recommended in patients with suspected PM, it is an invasive procedure that might increase the risk of infection [8]. As for conventional imaging, it is difficult to detect and evaluate peritoneal metastases, especially occult metastases, which presents challenges for early diagnosis and selection of appropriate treatment for patients. Notably, these modalities lack clear predictive value for the short-term prognosis of PM [9]. An increasing array of research suggests that PM should be regarded as a unique disease entity requiring tailored treatment strategies, beyond just a secondary manifestation of their primary tumors [1, 3]. There is an urgent need for diagnostic methods that enable early depiction of PM and predict outcomes, which are important for treatment decision-making and improving quality of life.

^18^F-fuorodeoxyglucose positron emission tomography/computed tomography (^18^F-FDG PET/CT) is characterized as noninvasive and repeatable, and acknowledged as a vital role in cancer staging and therapeutic monitoring [10, 11]. However, ^18^F- FDG PET/CT has demonstrated low to moderate sensitivity for PM, likely due to the strong desmoplastic reaction characterized by a strong desmoplastic reaction in histopathology [12]. The cancer-associated fibroblasts (CAFs) and extracellular fibrosis, which are important parts of the tumor microenvironment (TME), have been reported to account for up to 90% of the gross tumor mass, with tumor cells constituting a minority [13]. CAFs are involved in the promotion of tumor cell invasion, angiogenesis and growth, and correlate with a poor prognosis [14], necessitating reliable biomarkers for further exploration [15]. Studies confirm that the expression of α-smooth muscle actin (α-SMA) and fibroblast activation protein are important for studying the role of CAFs in cancers [16]. Fibroblast activation protein (FAP), a dipeptidyl peptidase 4 protein, has limited expression in normal adult tissues, but is overexpressed on CAFs in a variety of tumor stroma of many epithelial tumors and can specifically target tumor stroma [17]. In the earlier studies, FAP was reported to be a promising pan-cancer diagnostic and therapeutic target. FAP Inhibitors (FAPI) specifically targeting FAP exhibit excellent pharmacokinetic parameters, enhancing the feasibility of FAP as an imaging and therapeutic target. Radiolabeled FAPI compounds (e.g., ^68^Ga-FAPI and ^18^F-FAPI-04) PET/CT, have been certified to be superior to ^18^F-FDG PET/CT in the detection of peritoneal involvement with high image quality [18, 19]. Nevertheless, ^68^Ga is limited by the relatively short half-life and the need for ^68^Ge/^68^Ga generator [20]. By contrast, ^18^F offers advantages including scalable production, lower positron energy leading to better spatial resolution, and appropriate half-life. And there have already been reported that ^18^F-FAPI has a comparable lesion detection rate to ^68^Ga-FAPI [21]. Although, ^18^F-FAPI PET/CT was regarded able to detect PM with high sensitivity, the uptake characteristics of ^18^F-FAPI in different types of peritoneal carcinomatosis comparing to ^18^F-FDG and its relationship with the expression of FAP have not been systematically investigated. The predictive and prognostic value of ^18^F-FAPI-04 FAPI PET/CT has been proved in certain tumors [22], but there is lack of research on the value of ^18^F-FAPI PET/CT for clinical management in end-stage malignancy with PM.

This investigation aims to describe the characteristics of ^18^F-FAPI-04 PET/CT of PM in different types of primary malignant tumors by comparing with those in ^18^F-FDG PET/CT, and preliminary explore the relationship between ^18^F-FAPI-04 PET/CT findings and the expression of FAP. And to evaluate the value of ^18^F-FAPI-04 PET/CT in predicting short-term outcome of PM in end-stage malignancy after chemotherapy as well.

## Materials and methods

### Patients

We retrospectively analyzed 39 patients referred to our department from August 2021 to June 2023 to determine the PET/CT imaging features of PM. These patients underwent PET/CT scans due to clinically high suspicion of peritoneal metastasis for further evaluation. Some had previously received treatment for primary tumors, while others were newly diagnosed with peritoneal metastasis. Inclusion Criteria: (1) Patients with pathologically confirmed or clinically highly suspected PM, regardless of prior treatment for the primary tumor, but need to be more than 1 year since last treatment; (2) Patients referred to our center by clinicians for pre-treatment ^18^F-FAPI-04 and ^18^F-FDG PET/CT scans (both scans completed within one week) to evaluate tumor burden, detect metastases, and restage the disease; (3) Patients scheduled to receive pathological biopsy and treatment such as chemoradiotherapy; (4) Availability of comprehensive clinical data, including medical history, abdominal enhanced abdominal CT(CE-CT), and laboratory tests. Exclusion Criteria: (1) Patients with other distant metastases, concurrent other malignancies, or severe comorbidities; (2) Incomplete or missing clinical and imaging data; (3) Lack of subsequent treatment or incomplete follow-up. Blood tests before treatment, including carcinoembryonic antigen (CEA) and carbohydrate antigen 125(CA-125) levels, were collected. This retrospective analysis was approved by the Institutional Ethics Committee of the First Affiliated Hospital, School of Medicine, Zhejiang University. Informed written consent was provided to all participants before their participation in this study.

### PET/CT imaging

The Al^18^F-NOTA-FAPI-04 was synthesized following a previously reported method [23], with ^18^F produced by a cyclotron (Siemens Eclipse) and NOTA-FAPI-04 precursor obtained from Allinone (BEIJING PET TECHNOLOGY CO., LTD). The radiochemical purity of both ^18^F-FAPI-04 and ^18^F-FDG was more than 95%. Imaging with ^18^F-FAPI-04 and ^18^F-FDG was performed approximately 60 min after intravenous administration of each tracer (3.7 MBq/kg) using a PET/CT scanner (Siemens Biograph Vision). Patients underwent static PET/CT scans from the skull vertex to the mid-femur after bladder voiding. For anatomical localization and attenuation correction, low-dose CT imaging was conducted using the following parameters: tube current 150 mAs, voltage 120 kV, and a 512 × 512 acquisition matrix. Subsequent PET acquisition employed a 3D mode with 3-minute per-bed-frame duration and a 200 × 200 reconstruction matrix. Image reconstruction utilized the TrueX time-of-flight algorithm, configured with 2 iterative updates, 21 subsets, and a 4-mm Gaussian smoothing kernel, generating 3-mm-thick slices in a 440 × 440 matrix format. All post-processing operations were executed on a Syngo.via Client 4.1 platform (Siemens Healthineers, USA).

### Imaging evaluation

The images were reviewed independently by two experienced nuclear medicine physicians after being processed on the Siemens workstation (syngo. via). Any discordant results were decided through consultation. The maximum standardized uptake values (SUV_max_) of the most avid peritoneal lesions, liver and mediastinum blood pool were quantified by delineating regions of interest (ROIs) directly on the PET images. To ensure comparability, the tumor-to-liver ratio (T/L) and tumor-to-mediastinum blood pool ratio (T/B) were calculated using the following formulas: T/L = Peritoneal lesions SUV_max_/Liver SUV_max_, T/B = Peritoneal lesions SUV_max_/Mediastinum blood pool SUV_max_.

### Immunohistochemistry

The expression of FAP and α-SMA in cancer tissues was examined by immunohistochemistry (IHC) using FAP antibody (BOSTER Biological Technology Co., Ltd) or α-SMA antibody (Servicebio Technology Co., Ltd, GB13044), both diluted at 1:200. Semi-quantitative IHC scoring were performed under a light microscope at ×100 magnification by specifying percentage of positive cells and staining intensity by pathologists blinded to clinical data. FAP expression was scored based on staining intensity and the percentage of FAP-positive cells. Staining intensity was graded as: 0 (absent staining), 1 (weak staining), 2 (moderate staining), and 3 (strong staining). The percentage of positive cells was categorized into five groups: 0 (0–10%), 1 (11–25%), 2 (26–50%), 3 (51–75%), and 4 (76–100%).

### Evaluation criteria

The abdominal CE-CT scans performed before treatment and during the 6-week follow-up were collected. These CE-CT scan results were independently reviewed by two experienced imaging specialists and used to assess the short-term outcomes of the patients at 6 weeks after chemotherapy, according to the Response Evaluation Criteria in Solid Tumors (RECIST) version 1.1. Measurable lesions were selected as target lesions for recording the location and size of PM on axial CE-CT images, and the evaluation of those lesions that are not measurable was conducted as non-target lesions. After receiving therapy in accordance with National Comprehensive Cancer Network (NCCN) guidelines, patients were classified as responders with an outcome of complete or partial response, and non-responders with an outcome of stable or progressive disease, according to the RECIST criteria (v.1.1).

### Statistical analysis

SPSS software (version 23.0, IBM Inc.) was used for statistical analysis. Frequencies and percentages were utilized to describe categorical variables. Continuous data but non-normal distribution were presented as median (P25, P75). The Mann-Whitney U test was employed to compare uptake parameters, including SUV_max_, T/L and T/B, between the ^18^F-FAPI-04 and ^18^F-FDG groups. The Wilcoxon signed-rank test was used for within-group comparisons. Pearson’s correlation test was performed for correlation analysis. Logistic regression analysis was used to evaluate associations between clinical/imaging characteristics and short-term outcomes of PM. Receiver operating characteristic (ROC) curve analysis was used for SUV_max_, T/L and T/B in distinguishing responders and non-responders. Two-sided P-values < 0.05 were considered statistically significant.

## Results

### Patients characteristics

Thirty-nine patients were enrolled in this study, with a median age of 62 years (range 31–86 years). Based on histopathology, these patients were categorized into four different types: pancreatic cancer (17 cases, 43.59%), cholangiocarcinoma (8 cases, 20.51%), gastric cancer (6 cases, 15.38%), and colorectal cancer (8 cases, 20.51%). The characteristics of the patients are summarized in Table 1. All patients received four to eight cycles of chemotherapy, among whom six also underwent radiotherapy or intraperitoneal perfusion therapy. After 6 weeks of follow-up for short-term outcomes, 17 patients responded, whereas 22 did not.

**Table 1:** Patients characteristics

Factors	Category	Total number of cases(%)
Gender	Male	23(58.97)
	Female	16(41.03)
Age	≥ 65	18(46.15)
	< 65	21(53.85)
Pathological types	Pancreatic cancer	17(43.59)
	Cholangiocarcinoma	8(20.51)
	Gastric cancer	6(15.38)
	Colorectal cancer	8(20.51)
Previous Therapy	No	19(48.72)
	Yes	20(51.28)
CEA (µg/L)	≤ 5.0 µg/L	21(53.85)
	> 5.0 µg/L	18(46.15)
CA-125(U/ml)	≤ 35 U/ml	15(38.46)
	> 35 U/ml	24(61.54)
Clinical symptoms	Abdominal distention and pain	15(38.46)
	Diarrhea	7(17.95)
	Nausea and vomiting	9(23.08)
	Inappetence	8(20.51)
Types of PM	Localized	16(41.02)
	Diffuse	23(58.97)
Seroperitoneum	No	24(61.54)
	Yes	15(38.46)
End-stage therapy	Gemcitabine based	5(12.82)
	5-Fluorouracil based	9(23.08)
	Oxaliplatin based	11(28.20)
	Combination	14(35.90)

### The difference in ^18^F-FAPI-04 and ^18^F-FDG PET uptake of PM lesion

**Fig. 1:**
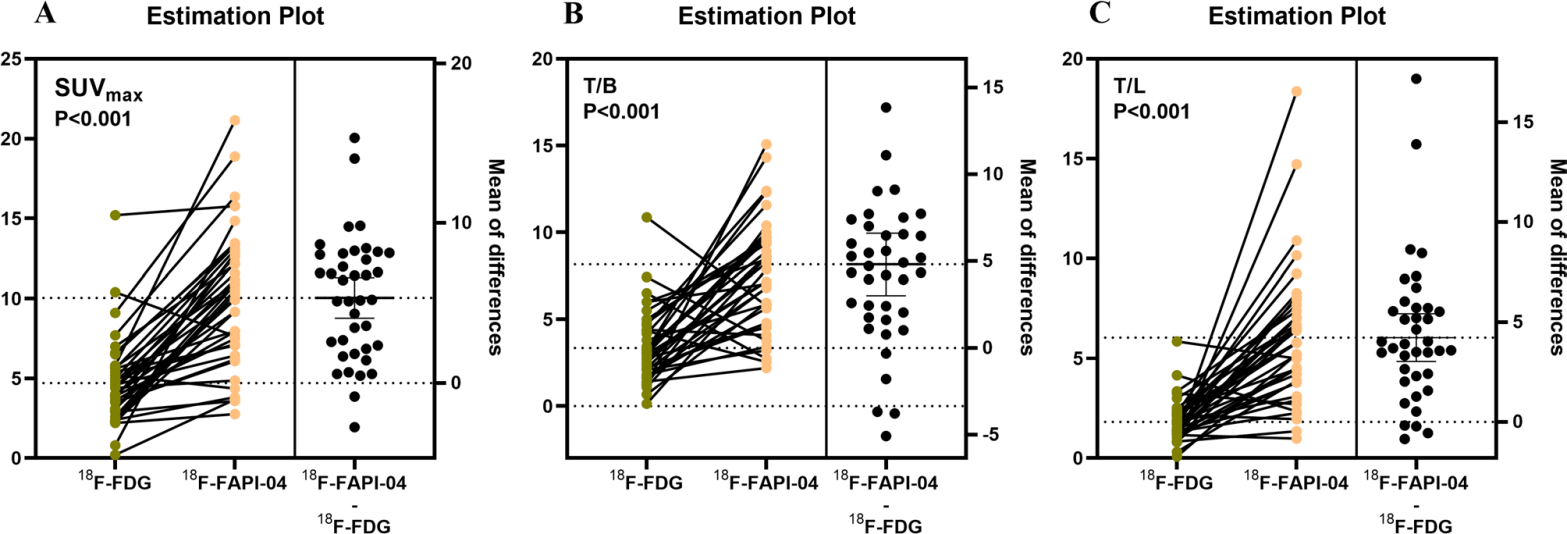
Estimation plots of ^18^F-FDG and ^18^F-FAPI-04 uptake parameters in PM. The SUV_max_(**A**), T/L (**B**) and T/B (**C**) of PM in ^18^F-FAPI-04 PET/CT were significantly higher than those in ^18^F-FDG PET/CT

**Fig. 2:**
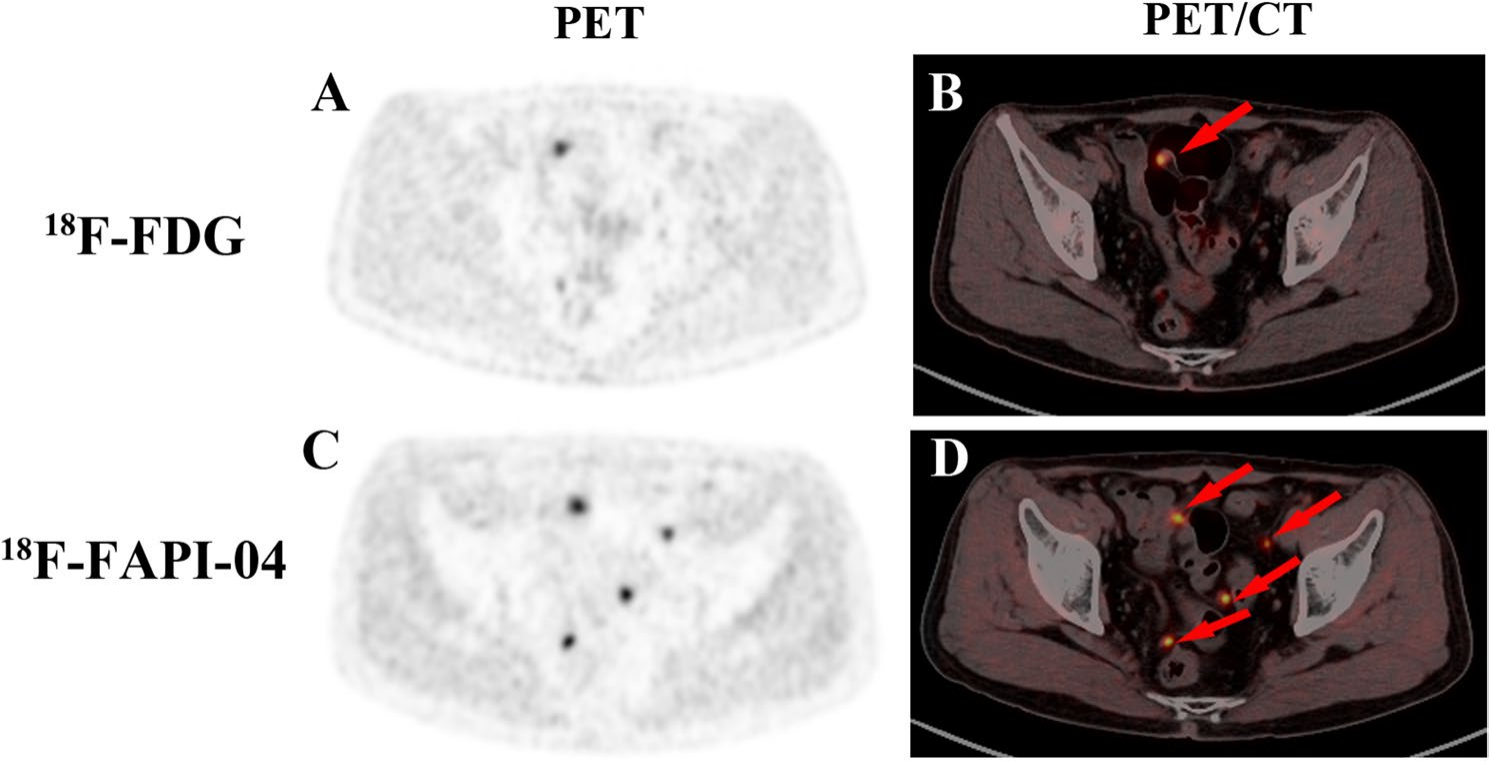
A 62-year-old male patient with pancreatic cancer-associated PM underwent PET/CT imaging for evaluation. ^18^F-FAPI-04 PET exhibited markedly higher uptake in pelvic lesions than ^18^F-FDG PET and identified additional metastatic foci

**Fig. 3:**
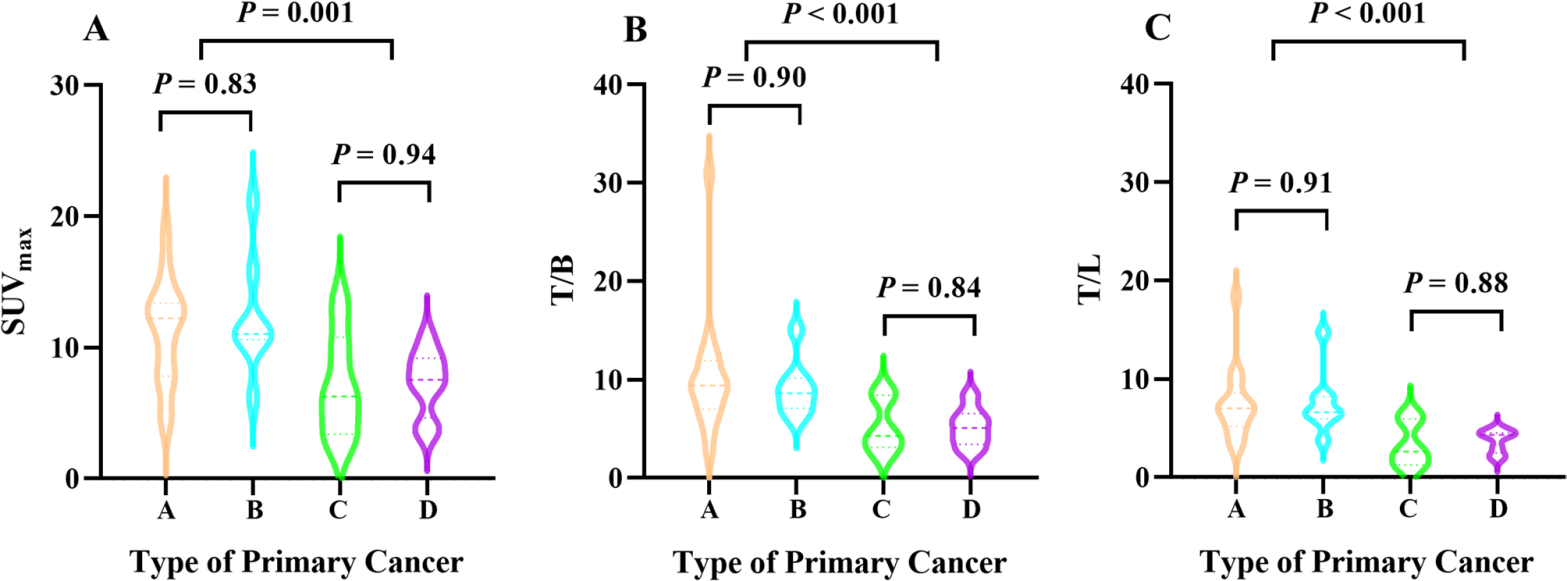
Violin plots of ^18^F-FAPI-04 uptake parameters in four different pathological types of PM. **A**: pancreatic cancer, **B**: cholangiocarcinoma, **C**: gastric carcinoma, **D**: colorectal cancer. The Kruskal-Wallis test showed that the difference in ^18^F-FAPI-04 uptake among PM of different pathological types were statistically significant (*P* < 0.05)

**Fig. 4:**
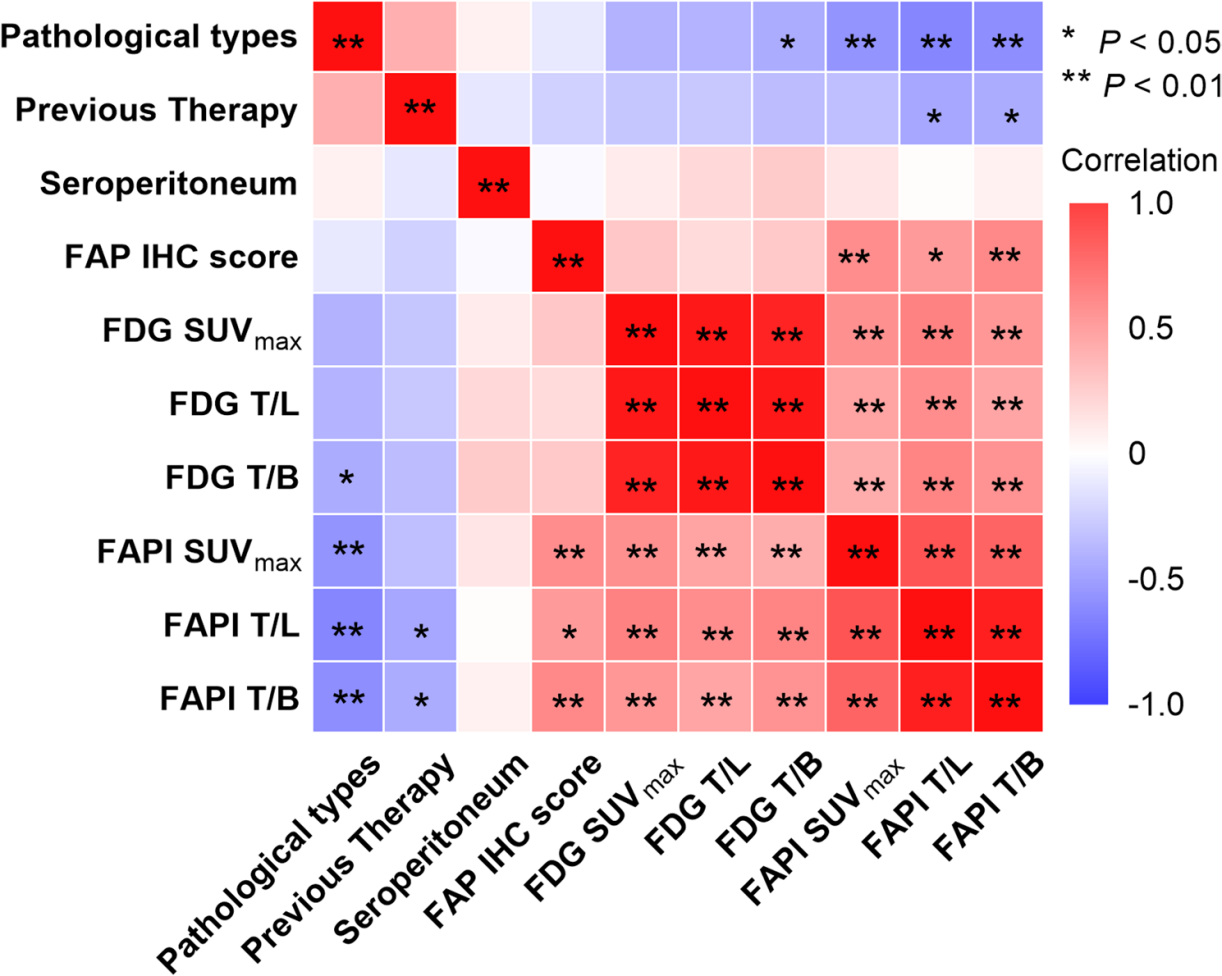
Correlogram showing Spearman correlation coefficients between ^18^F-FAPI-04 PET/CT and ^18^F-FDG PET/CT uptake variables and clinical variables in patients with peritoneal metastasis. Red indicates positive correlations between two variables, and blue indicates negative correlations. Darker colors indicate stronger correlations

The SUV_max_, T/L and T/B of PM lesions on ^18^F-FAPI-04 PET/CT were 10.30 (7.07,12.83), 5.78 (3.79,7.37) and 7.86 (4.79,9.71), respectively, compared to 4.40 (3.00,5.60), (1.66 (1.18,2.19) and 3.00 (2.10,4.40) on ^18^F-FDG PET/CT. The Mann-Whitney U test demonstrated significantly higher uptake parameters for ¹⁸F-FAPI-04 than ¹⁸F-FDG (Z = -5.19, *P* < 0.001; Z = -5.34, *P* < 0.001; Z = -5.11, *P* < 0.001, respectively) (Fig. 1***and*** Fig. 2***)***. For different cancer types, the SUV_max_ of PM lesions on ¹⁸F-FAPI-04 PET/CT in pancreatic cancer, cholangiocarcinoma, gastric cancer, and colorectal cancer was 12.23 (7.80,13.38), 11.05(10.62,14.71), 6.26(5.96,8.22) and 7.54(4.63,9.18), respectively. T/L and T/B values also varied significantly across cancer types (all *P* < 0.05). Further analysis revealed higher uptake parameters in pancreatic cancer and cholangiocarcinoma compared to gastric and colorectal cancers (all *P* < 0.05), but no differences were observed between pancreatic cancer and cholangiocarcinoma (Z = -0.058, *P* = 0.98 for SUV_max_; Z = -1.40, *P* = 0.18 for T/L and Z = -0.55, *P* = 0.59 for T/B) or between gastric and colorectal cancers (Z = -0.074, *P* = 0.94 for SUV_max_, Z = -0.16, *P* = 0.88 for T/L and Z = 0.20, *P* = 0.84 for T/B). Not unexpectedly, significant differences were found between pancreaticobiliary cancer (pancreatic cancer and cholangiocarcinoma) and gastroenteric cancer (gastric cancer and colorectal cancer) (Z =-3.31, *P =* 0.001 for SUV_max_, Z = -4.01, *P* < 0.001 for T/L and Z = -3.78, *P* < 0.001 for T/B) **(**Fig. 3***and*** Table 2***)***. In contrast, the ¹⁸F-FDG uptake parameters among different cancer types were not statistically significant for SUV_max_ (Z = 5.08, *P* = 0.166), T/L (Z = 6.05, *P* = 0.109), and T/B (Z = 6.50, *P* = 0.090). Any previous therapy before ^18^F-FAPI-04 PET PET/CT has no influence on the uptake parameter (Z = -1.813, *P* = 0.072 for SUV_max_, Z = -1.22, *P* = 0.23 for T/L, and Z = -2.18, *P* = 0.29 for T/B). The correlation analysis indicated that ^18^F-FAPI-04 uptake parameters, including SUV_max_, T/L, and T/B, were associated with pathological types (*r* = -0.43, *P* = 0.007; *r* = 0.41, *P* = 0.01 and *r* = 0.41, *P* = 0.01, respectively), whereas ^18^F-FDG uptake parameters showed no significant correlation with pathology but were positively correlated with ^18^F-FAPI-04 uptake parameters (Fig. 4). The uptake parameters of ^18^F-FAPI-04 and ^18^F-FDG PET, as well as the pathological tumor types, were not significantly associated with the presence of malignant ascites, CEA levels, or CA-125 values (all *P* > 0.05).

**Table 2:** 18 F-FAPI-04 and 18 F-FDG PET uptake parameters of different pathological types of PM

	Types of cancer (Median (P25, P75))	SUV_max_	T/L	T/B
^18^F-FAPI-04 PET	Pancreatic cancer	12.23 (7.80,13.38)	7.00 (5.19,8.60)	9.43(6.99,11.94)
	Cholangiocarcinoma	11.05(10.62,14.71)	6.62(5.96,8.22)	8.61(7.07,10.18)
	Gastric carcinoma	6.26(3.38,10.78)	2.63(1.26,5.95)	4.28(3.11,8.41)
	Colorectal cancer	7.54(4.63,9.18)	4.33(2.47,4.52)	5.09(3.43,6.56)
^18^F-FDG PET	Pancreatic cancer	5.00(3.85,6.80)	2.08(1.40,2.49)	4.00(2.59,5.25)
	Cholangiocarcinoma	4.35(2.83,5.78)	1.53(1.19,2.18)	2.59(1.77,3.17)
	Gastric carcinoma	3.30(2.73,4.85)	1.44(1.11,1.76)	2.72(2.07,3.51)
	Colorectal cancer	3.80(2.33,4.85)	1.27(0.79,1.73)	1.95(1.14,3.00)

### The correlation between ^18^F-FAPI-04 PET uptake and histological FAP expression in PM lesions of different pathology

**Fig. 5:**
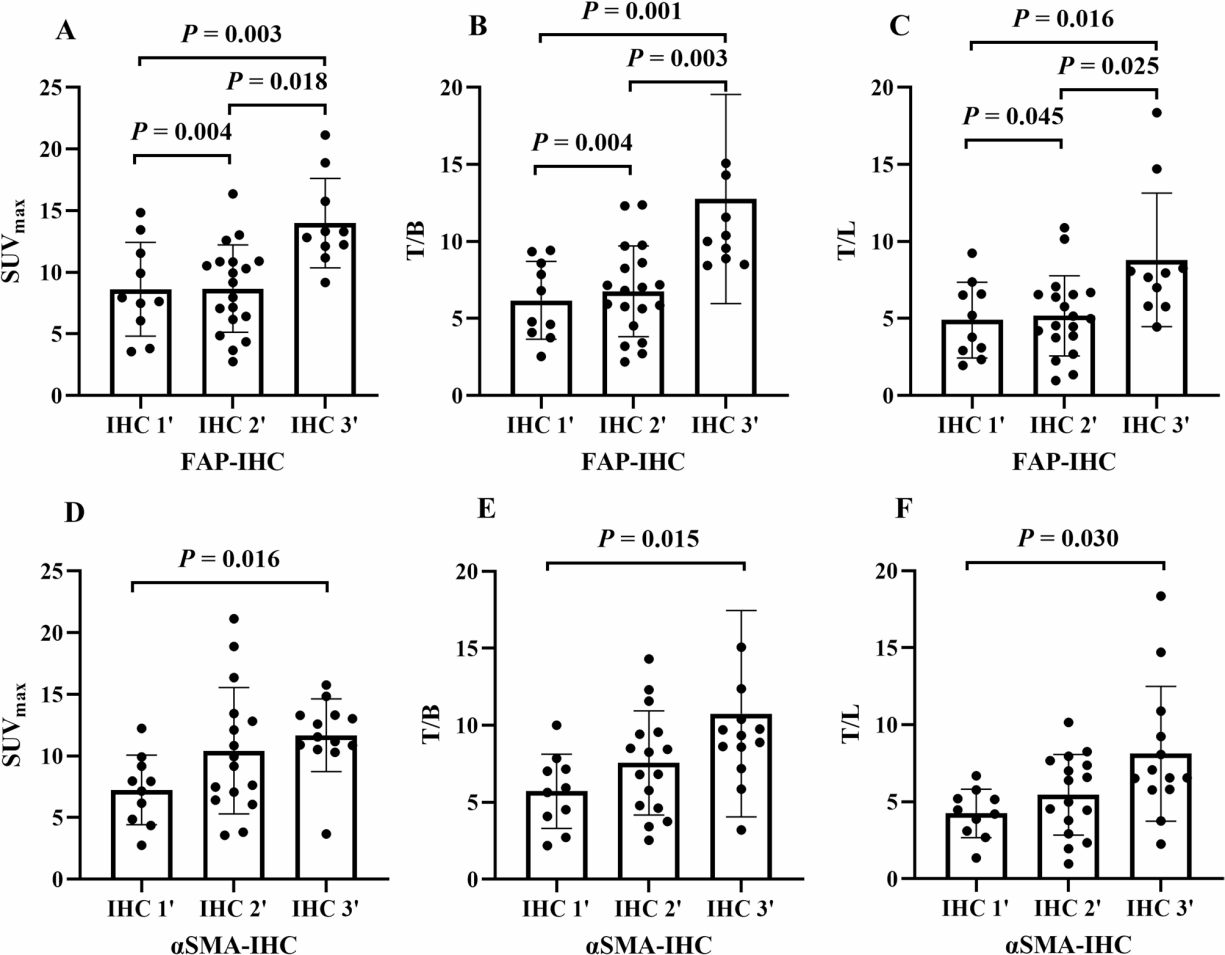
Comparison of ^18^F-FAPI-04 Uptake Parameters Across FAP-IHC and α-SMA-IHC Score Groups. Bar plots demonstrate significant differences in ^18^F-FAPI-04 uptake parameters among groups with FAP-IHC scores 1, 2, and 3 (**A-C**), **A**: SUV_max_; **B**: T/B; **C**: T/L. Bar plots show no statistically significant differences in ^18^F-FAPI-04 uptake parameters across α-SMA-IHC score groups 1, 2, and 3(**D-F**), **A**: SUV_max_; **B**: T/B; **C**: T/L

Elevated FAP and α-SMA expression were detected in all 39 peritoneal metastasis specimens (FAP scores: 0’_none = 0, 1’_mild = 10, 2’_moderate = 19, and 3’_intense = 10; α-SMA scores: 0’_none = 0, 1’_mild = 10, 2’_moderate = 16, and 3’_intense = 13). The FAP IHC score showed significant positive correlations with the SUV_max_, T/L and T/B of ^18^F-FAPI-04 respectively (r = 0.46, *P* = 0.003, r = 0.41, *P* = 0.009 and r = 0.49, *P* = 0.002). Consistent with this, the SUV_max_, T/L, and T/B of ^18^F-FAPI-04 increased progressively with higher FAP IHC scores. Specifically, the SUV_max_ values for FAP IHC 1’, 2’, and 3’ groups were 7.79 (5.52, 12.03), 9.17 (6.18, 10.87), and 13.07 (11.89, 16.55), respectively; T/L values were 4.50 (2.78, 6.79), 4.99 (3.76, 6.56), and 7.82 (5.80, 9.87); and T/B values were 5.80 (4.00, 8.78), 6.82 (4.53, 8.63), and 10.20 (8.79, 14.50) (all *P* < 0.05; Fig. 5).

**Fig. 6:**
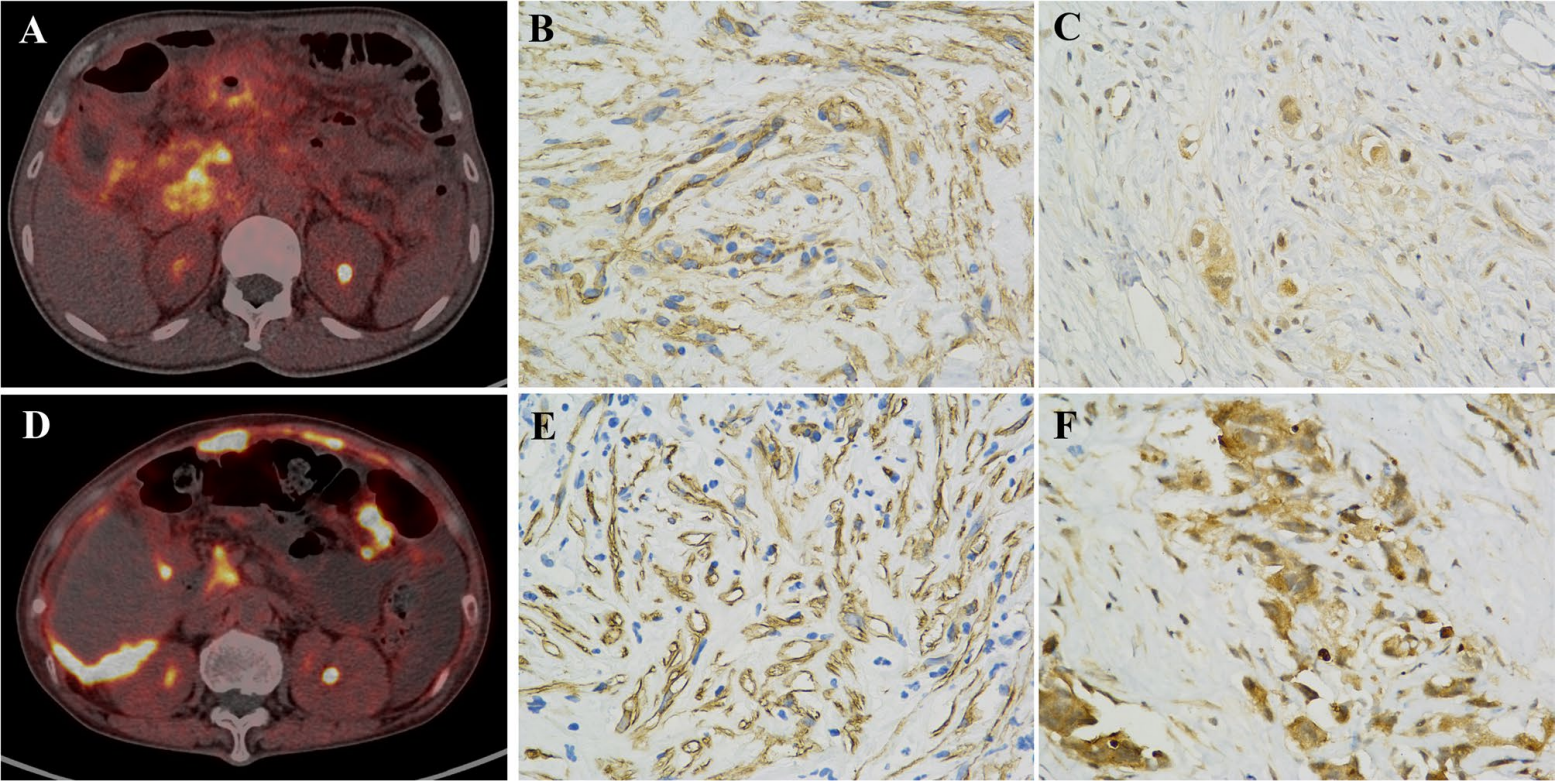
^18^F-FAPI PET/CT imaging and FAP immunohistochemical results (×400, DAB staining) in peritoneal metastases of pancreatic and gastric cancers. **A**: ^18^F-FAPI-04 PET/CT of gastric cancer, **B**: α-SMA expression in gastric cancer, **C**: FAP expression in gastric cancer, **D**: ^18^F-FAPI-04 PET/CT of pancreatic cancer, **E**: α-SMA expression in pancreatic cancer, **F**: FAP expression in pancreatic cancer. FAP expression in pancreatic cancer was higher than that in gastric cancer

Similarly, the α-SMA IHC score was significantly associated with the SUV_max_, T/L and T/B of ^18^F-FAPI-04 PET respectively (r = 0.395, *P* = 0.013, r = 0.45, *P* = 0.005; r = 0.40, *P* = 0.011). Notably, SUV_max_, T/L, and T/B values in the high α-SMA score groups were significantly higher than those in low α-SMA score groups. The SUV_max_, T/L, and T/B values across α-SMA IHC 1’, 2’, and 3’ groups were as follows: 7.55 (4.74, 9.36), 9.56 (6.59, 13.29) and 11.56 (10.71, 13.32) for SUV_max_, 4.34 (3.00, 5.35), 5.69 (3.14, 7.60), 6.56 (5.79,10.07) for T/L, and 5.79(3.75,7.34), 7.54(4.67,9.52), 9.34(7.89,11.39) for T/B respectively (*P* < 0.05, Fig. 5). Immunohistochemical staining further suggested that α-SMA and FAP expression in lesions with high ^18^F-FAPI-04 uptake was significantly higher than in lesions with low ^18^F-FAPI-04 uptake (Fig. 6).

### The correlation between ^18^F-FAPI-04 PET uptake and different short-term outcomes in PM lesions

**Fig. 7:**
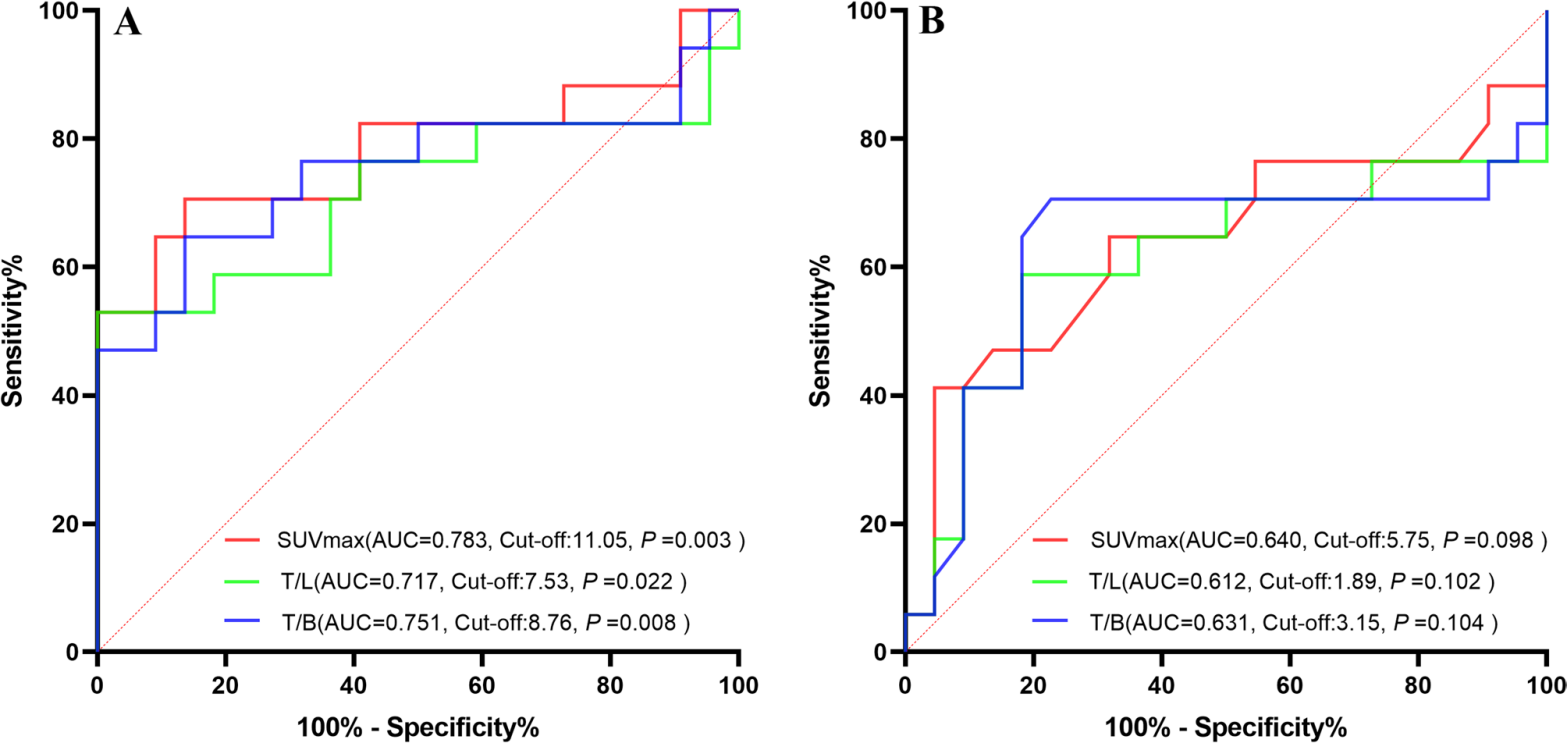
Receiver operating characteristic (ROC) curve analyses of SUV_max_, T/L, and T/B cutoff values demonstrated their significance as predictors of short-term outcomes in PM. **A**: ^18^F-FAPI-04 PET/CT parameters, **B**: ^18^F-FDG PET/CT parameters, *P < 0.05; **P < 0.01

A positive correlation was observed between ^18^F-FAPI-04 uptake parameters and worse short-term outcomes (*P* < 0.001); however, no significant correlation was found between short-term outcomes and ^18^F-FDG uptake parameters (Table 3). The SUV_max_, T/L, and T/B of ^18^F-FAPI-04 in responders and non-responders showed statistically significant differences (Z = -3.00, *P* = 0.002; Z = -2.29, *P* = 0.021; Z = -2.66, *P* = 0.007), while no comparable differences were observed for ^18^F-FDG (Table 4). No significant correlation was found between short-term outcomes of PM and clinical factors such as pathological type or prior treatment history (all *P* > 0.05). The optimal cut-off values of ^18^F-FAPI-04 uptake parameters for distinguishing treatment response were as follows: SUV_max_ = 11.05, T/L = 7.53, and T/B = 8.76. The corresponding AUC values were 0.783, 0.717, and 0.751, with sensitivity and specificity of 70.60% and 80.40%, 58.82% and 81.82%, 64.71% and 86.37%, respectively, while ^18^F-FDG showed no predictive value for treatment response (Fig. 7). Univariate and multivariate logistic regression analyses identified SUV_max_ as a significant predictor of short-term outcomes in PM, with higher SUV_max_ values indicating worse outcomes (*P* = 0.033; OR = 1.35; 95% CI: 1.03–1.79). Neither ^18^F-FDG SUV_max_ nor other clinical factors showed significant predictive value for short-term outcomes (Table 5).

**Table 3:** Correlation analysis of clinical indicators and short-term outcome

Factors	Correlation	*P* value(*<0.05)
^18^F-FAPI SUV_max_	0.470	0.003*
^18^F-FAPI T/L	0.435	0.006*
^18^F-FAPI T/B	0.438	0.005*
^18^F-FDG SUV_max_	0.221	0.176
^18^F-FDG T/L	0.179	0.275
^18^F-FDG T/B	0.230	0.159
Gender	0.208	0.205
Age	-0.127	0.439
Previous Therapy	-0.061	0.710
Pathological types	-0.139	0.398
Seroperitoneum	0.097	0.558

**Table 4:** ^18^ F-FAPI-04 and 18 F-FDG uptake parameters of PM in different short-term outcome groups

Parameters (Median (P25, P75))		Responders	Non-responders	*P* value
^**18**^ **F-FDG**	**SUV**_**max**_	3.95 (2.98, 5.05)	5.00 (3.10, 6.80)	0.14
	**T/L**	1.47 (1.18, 1.84)	2.08 (1.01, 2.49)	0.24
	**T/B**	2.59 (2.10, 3.11)	3.80 (1.46, 5.25)	0.17
^**18**^ **F-FAPI-04**	**SUV**_**max**_	7.95 (6.16, 10.85)	13.03 (9.55, 15.31)	0.002**
	**T/L**	4.77 (3.60, 6.55)	7.68 (4.84, 9.70)	0.021*
	**T/B**	6.38 (4.60, 8.52)	9.55 (7.34, 12.35)	0.007*

* *P* < 0.05, ** *P* < 0.01

**Table 5:** The correlation between clinical and imaging features and short-term outcome of peritoneal metastasis was analyzed using logistic regression analysis

Factors	Univariate analysis			Multivariate analysis		
	*P* value	OR	95%CI	*P* value	OR	95%CI
FAPI SUV_max_	0.009*	1.334	1.074 ~ 1.657	0.033*	1.354	1.025 ~ 1.788
FDG SUV_max_	0.193	1.201	0.912 ~ 1.582	0.730	1.056	0.775 ~ 1.440
Gender	0.199	0.417	0.109 ~ 1.586	0.411	0.510	0.103 ~ 2.538
Age	0.430	0.979	0.928 ~ 1.032	0.615	0.985	0.929 ~ 1.045
Previous Therapy	0.701	1.284	0.358 ~ 4.602	0.559	0.611	0.117 ~ 3.193
Pathological types	0.460	0.602	0.157 ~ 2.311	0.777	1.115	0.525 ~ 2.365
Seroperitoneum	0.547	0.667	0.178 ~ 2.491	0.763	0.777	0.152 ~ 3.982

## Discussion

This study demonstrates that the uptake parameters of ^18^F-FAPI-04 PET/CT in PM lesions are not only higher than those of ^18^F-FDG PET/CT but also exhibit distinct ^18^F-FAPI-04 uptake patterns, which correlate with the expression levels of FAP in the pathology of PM. More importantly, the study evaluates the predictive value of ^18^F-FAPI-04 PET/CT for short-term outcomes following chemotherapy in patients with peritoneal metastases as a distinct disease entity.

PM has limited treatment options and a poor prognosis owing to late diagnosis and limited response to systemic chemotherapy [24]. To explore effective therapeutic approaches for peritoneal metastatic tumors, researchers have identified multiple molecular mechanisms underlying the poor prognosis associated with this condition [25, 26]. A growing number of researchers emphasize the impact of the TME on the treatment response of peritoneal metastasis [27]. Revealing the molecular characteristics of PM and its interactions with the TME is critical to understanding its resistance to existing therapies, which may provide insights into optimizing current regimens and developing novel targeting strategies [28]. CAFs, which are found in both primary and metastatic tumors, are multifunctional cells that participate in cancer progression through complex interactions with other cell types in the TME [16]. They were implicated in promoting tumor cell invasion, angiogenesis and growth and their presence correlates with a poor prognosis [29, 30]. Although CAFs are considered attractive diagnostic and therapeutic targets, their precise role in tumor progression remains controversial [31]. FAP, which is overexpressed in CAFs, is one of the most classical markers to study the relationship between CAFs and tumor progression. Radionuclide-labeled FAPI has been extensively studied in cancer imaging and showed better detection rates than ^18^F-FDG PET/CT [32, 33]. A systematic review highlighted that the diagnostic sensitivity of ^68^Ga-FAPI for PM is significantly higher than that of ^18^F-FDG PET/CT, which is helpful for the formulation of surgical plans and the selection of patients, and that ^68^Ga-FAPI PET/CT is particularly beneficial for the detection of smaller lesions and lesions located next to abdominal structures with high physiological tracer uptake [34]. However, these findings lack robust pathological validation. As consistent with the literature reports, this study demonstrated that pathologically confirmed PM lesions exhibited significantly higher ^18^F-FAPI-04 uptake parameters than those of ^18^F-FDG. These findings suggest that ^18^F-FAPI-04 PET/CT not only detects PM with higher sensitivity compared to ^18^F-FDG PET/CT but also may enhance the accuracy of PM assessment, thereby providing stratification criteria for personalized treatment strategies. This enhanced ^18^F-FAPI-04 uptake may be attributed to the high expression of FAP in the predominant cell population within the tumor stroma of many epithelial malignancies (known as CAFs), combined with the observation that most PMs histopathologically exhibit a pronounced desmoplastic reaction, while the original tumor cells constitute a minority [35], leaving original tumor cells in the minority.

Kratochwil, C., et al. observed that ^68^Ga-FAPI uptake decreased sequentially in cholangiocarcinoma, colorectal, pancreatic, and gastric cancer [12]. They reported that cholangiocarcinoma exhibited a higher mean uptake value of ^68^Ga-FAPI, with SUV_max_ exceeding 12, while colorectal cancer and pancreatic cancer showed moderate SUV_max_ values (6–12), and gastric cancer demonstrated a mean uptake value below 6. These findings differ from our observed SUV_max_ values, which may be attributed to differences in tumor biodistribution caused by structural variations between the ^68^Ga-FAPI and ^18^F-FAPI tracers [21]. Alternatively, the discrepancy could stem from the fact that, unlike previous studies focusing on primary tumors, our analysis targeted peritoneal metastases. Heterogeneity during peritoneal dissemination might result in metastatic lesions containing richer fibroblast-associated stromal components compared to primary tumors [36, 37]. In this study, there was no difference in the uptake of ^18^F-FAPI-04 by PMs of pancreatic cancer, cholangiocarcinoma, gastric cancer, and colorectal cancer. However, significant differences were observed when these cancers were classified into pancreatobiliary and gastrointestinal categories. But the accordant uptake differences were not found in ^18^F-FDG imaging. The different uptake of ^18^F-FAPI-04 might be related to the fact that the primary cancer promotes a different degree of desmoplastic reaction in PM tissues. In accordance with previously reported, FAP was highly expressed in cancer cells and fibroblasts of pancreatic cancer tissues, and its high expression was associated with desmoplasia and results in poor prognosis [38]. A recent investigation revealed that pancreatic cancer exhibits high FAPI avidity, with immunohistochemical staining confirming that this uptake directly correlates with tumoral FAP expression levels [39]. Mona, C.E., et al. [40] studied the spectrum of FAP expression across various cancers by immunohistochemistry and indicated that strong FAP expression was observed in cancers of the bile duct, colon, stomach, and pancreas. They also revealed that the biodistribution of ^68^Ga-FAPI PET in these cancers correlates with FAP expression. In the present study, IHC staining was performed on the PM of these 39 patients. The results demonstrated that the IHC scores of FAP and α-SMA were significantly correlated with the uptake of ^18^F-FAPI-04, further indicating that ^18^F-FAPI-04 serves as an effective imaging modality for in vivo visualization of lesion fibrosis and CAF infiltration.

Meanwhile, previous studies have reported on the relationship between prognosis or short-term outcome and ^18^F-FAPI PET, suggesting that high tumor uptake values and large uptake volumes are associated with poor prognosis and treatment efficacy [41]. However, there is a lack of studies on the relationship between ^18^F-FAPI-04 uptake in PM and short-term outcomes. Studies of ^18^F-FAPI-04 PET imaging have demonstrated TME activity associated with peritoneal metastases, which can aid in characterizing disease features, predicting and optimizing patient management, and providing independent prognostic information. In contrast, ^18^F-FDG PET imaging provides tumor metabolic profiles for tumor characterization and prognosis prediction. Interestingly, this study verified that ^18^F-FAPI-04 PET could be useful for predicting treatment efficiency of patients with PM regardless of the primary cancer type. The results indicated that higher ^18^F-FAPI uptake was associated with less favorable short-term outcomes, suggesting that FAP overexpression in CAFs within the TME may hold critical clinical significance for predicting poor prognosis in PM. The short-term outcomes of patients with PM were no significant correlated with ^18^F-FDG uptake parameters, consistent with previous reports that some cancers with poor prognostic often present with low uptake of ^18^F-FDG [42, 43] and also demonstrates that glucose metabolism do not fully describe cancer behavior. This further demonstrates that glucose metabolism does not fully characterize tumor behavior. Therefore, ^18^F-FAPI-04 PET/CT may compensate for the limitations of ^18^F-FDG PET/CT for clinical decision-making. Notably, SUV_max_ of ^18^F-FAPI-04 emerged as an independent factor affecting the short-term outcomes of patients with PM, suggesting that PM should be considered a distinct tumor entity. These findings support the conclusion that ^18^F-FAPI-04 serves as a valuable novel imaging biomarker for predicting and monitoring treatment responses.

Although the findings are encouraging, our study does have some limitations. Firstly, the limited number of patients may introduce statistical bias in the results. Second, some patients had previously undergone treatments such as surgery, making it impossible to collectively evaluate the primary lesions. Therefore, clinical trials with larger patient cohorts and more comprehensive surgical pathology data on primary tumors are needed. Finally, performing ^18^F-FAPI-04 PET imaging after treatment provides a more accurate assessment of therapeutic response, and future prospective studies are warranted to comprehensively explore these aspects.

## Conclusions

It was reasonably concluded that ^18^F-FAPI-04 PET imaging was effective in detecting the peritoneal metastasis tumors and predicting the short-term outcomes of systemic chemotherapy for patients with peritoneal metastasis.

## Data Availability

No datasets were generated or analysed during the current study.

## Abbreviations

TME: Tumor microenvironment
CAFs: Cancer-associated fibroblasts
α-SMA: Alpha-smooth muscle actin
FAP: Fibroblast activation protein
SUV_max_: Standardized uptake values
T/L: Tumor/liver ratio
T/B: Tumor/mediastinum blood pool ratio
PM: Peritoneal metastases
